# Fabrication of PEGylated Chitosan Nanoparticles Containing Tenofovir Alafenamide: Synthesis and Characterization

**DOI:** 10.3390/molecules27238401

**Published:** 2022-12-01

**Authors:** Muhammad Zaman, Muhammad Hammad Butt, Waqar Siddique, Muhammad Omer Iqbal, Naveed Nisar, Asma Mumtaz, Hafiza Yusra Nazeer, Abdulrahman Alshammari, Muhammad Shahid Riaz

**Affiliations:** 1 Faculty of Pharmacy, University of Central Punjab, Lahore 54000, Pakistan; 2 Department of Medicinal Chemistry, Faculty of Pharmacy, Uppsala University, 75123 Uppsala, Sweden; 3 Department of Pharmacy, University of South Asia (USA), Lahore 54000, Pakistan; 4 College of Pharmacy, University of Sargodha, Sargodha 40100, Pakistan; 5 Shandong Provincial Key Laboratory of Glycoscience and Glycoengineering, School of Medicine and Pharmacy, Ocean University of China, Qingdao 266003, China; 6 Royal Institute of Medical Sciences, Multan 59300, Pakistan; 7 Institute of Research and Advanced Studies of Pharmacy (IRASP), Multan 59300, Pakistan; 8 Faculty of Pharmacy, Bahauddin Zakariya University Multan, Multan 59300, Pakistan; 9 Multan Medical and Dental College, Multan 59300, Pakistan; 10 Department of Pharmacology and Toxicology, College of Pharmacy, King Saud University, Post Box 2455, Riyadh 11451, Saudi Arabia; 11 Nutrition and Food Science Area, Preventive Medicine and Public Health, Food Science, Toxicology and Forensic Medicine Department, Faculty of Pharmacy, Universitat de València, Avda, Vicent Andrés Estellés, s/n, Burjassot, 46100 València, Spain

**Keywords:** tenofovir alafenamide, antiretroviral drugs, polymer synthesis, chitosan, polyethylene glycol, nanocarrier system, nanoparticles

## Abstract

Tenofovir alafenamide (TAF) is an antiretroviral (ARV) drug that is used for the management and prevention of human immunodeficiency virus (HIV). The clinical availability of ARV delivery systems that provide long-lasting protection against HIV transmission is lacking. There is a dire need to formulate nanocarrier systems that can help in revolutionizing the way to fight against HIV/AIDS. Here, we aimed to synthesize a polymer using chitosan and polyethylene glycol (PEG) by the PEGylation of chitosan at the hydroxyl group. After successful modification and confirmation by FTIR, XRD, and SEM, TAF-loaded PEGylated chitosan nanoparticles were prepared and analyzed for their particle size, zeta potential, morphology, crystallinity, chemical interactions, entrapment efficacy, drug loading, in vitro drug release, and release kinetic modeling. The fabricated nanoparticles were found to be in a nanosized range (219.6 nm), with ~90% entrapment efficacy, ~14% drug loading, and a spherical uniform distribution. The FTIR analysis confirmed the successful synthesis of PEGylated chitosan and nanoparticles. The in vitro analysis showed ~60% of the drug was released from the PEGylated polymeric reservoir system within 48 h at pH 7.4. The drug release kinetics were depicted by the Korsmeyer–Peppas release model with thermodynamically nonspontaneous drug release. Conclusively, PEGylated chitosan has the potential to deliver TAF from a nanocarrier system, and in the future, cytotoxicity and in vivo studies can be performed to further authenticate the synthesized polymer.

## 1. Introduction

Antiretroviral (ARV) drugs are the cornerstone of HIV prevention and management. Since the advent of combination ARV therapy in 1995, ARV drugs have proven to be effective treatment options for the prevention of HIV infection [1]. Tenofovir is an ARV drug that belongs to the class of acyclic nucleoside phosphonates. Tenofovir has the potential to inhibit HIV, but an active metabolite (tenofovir diphosphate) has a strong inhibitory activity against HIV reverse transcriptase. In spite of these favorable properties, tenofovir in its parent form cannot be administered orally owing to its poor membrane permeability, poor in vitro drug release, and low oral bioavailability. Moreover, tenofovir has free ‘OH’ groups that easily ionize at intestinal pH, leading to limited permeability of the drug across the intestinal wall [2]. 

Tenofovir alafenamide (TAF) is a caspase-activated prodrug of tenofovir (TFV). Based on an in vitro analysis, the 50% effective concentration (EC50) of TAF is in the low nanomolar range (5 to 11.2 nM). TAF is intracellularly converted by kinases into tenofovir diphosphate, an active form of the drug that competitively inhibits HIV reverse transcriptase and the generation of viral transcripts [3]. The diphosphate, due to its charge and pKa, is highly impermeable to cellular membranes and is trapped in the intracellular volume compared to the parent species. TAF is a more potent prodrug than tenofovir diphosphate fumarate (TDF), resulting in low drug exposures and reduced side effects compared to TDF. Together, these characteristics make TAF one of the leading drug molecules for long-acting ARV delivery because it is potent and cellularly long-acting [4].

Biodegradable nanoparticle-based systems are capable of improving the therapeutic effects of bioactive molecules by enhancing their bioavailability, solubility, and retention time in the body. The encapsulation of drugs in polymeric nanoparticulate systems helps to improve drug efficacy, specificity, and tolerability as well their therapeutic indices. Traditional drug delivery systems and routes have numerous drawbacks, including the need to administer large drug doses to achieve the desired therapeutic response, the inability to reach target sites, and the failure to achieve desired therapeutic effects due to the interactions of drugs with biological molecules [5]. To overcome these problems, innovation has shifted towards nanoformulations for the purpose of achieving targeted drug delivery, the controlled or sustained release of drugs, the maintenance of therapeutic efficacy, and the avoidance of unwanted interactions. In this regard, nanoparticles can serve as efficient drug delivery systems to improve drug targetability, absorption, and bioavailability. Nanoparticles are colloidal particles based on solid materials, and they have sizes within the range of 1–1000 nm [6]. They allow control of the release pattern of drugs, and more than one drug can be incorporated in them to accomplish the aim of combination therapy [7]. Nanoparticles can be functionalized on the basis of different polymers, functional groups, ligands, and protein molecules to customize them according to the type of drug delivery required. Polymeric nanoparticles are the rising stars of novel drug delivery systems. Polymers are macromolecules formed by the union of a large number of monomers by covalent bonding in a linear or branched form. Many polymers exhibit the characteristics of biocompatibility, biodegradability, and nonimmunogenicity, which make them ideal candidates for use in the preparation of nanoparticles [8]. 

Chitosan is a water-insoluble linear amino polysaccharide obtained by the deacetylation of chitin. The biocompatible, biodegradable, nontoxic, and mucoadhesive properties of chitosan make it an ideal candidate for the formulation of polymeric nanoparticles. It has the ability to load both lipophilic and hydrophilic drugs and is quite inexpensive and readily available in the market. Chitosan also possesses a positive charge, which allows to react with negatively charged drugs and biomolecules, improving the absorption of drugs [9]. Moreover, chitosan has remarkable bioadhesive properties that make it suitable for use in mucoadhesive drug delivery systems aimed at enhancing the retention times of drugs by adhering to mucosae for longer periods of time. These properties render chitosan a favorable water-soluble natural polymer that is ideal for use in the preparation of polymeric nanoparticles [10]. 

Despite its amazing properties, the use of chitosan in drug delivery systems comes with certain problems. One of them is the limited solubility of chitosan at a pH value that is higher than its pKa value, i.e., pH 5.5–6.5. Chitosan exhibits good solubility in acidic conditions as a result of the protonation of the D-glucosamine units in its structure. In contrast, it shows poor or negligible solubility at neutral or alkaline pH values. Moreover, chitosan-based polymeric nanoparticles often suffer from the unwanted attacks of serum nucleases and proteins and undergo rapid clearance from blood circulation via the reticuloendothelial system (RES) [11]. This issue has again drawn attention from scientists hoping to modify the polymer and formulate long-acting polymeric nanoparticles. The literature reported that biocompatible polymers, such as polyethylene glycol, have the capacity to overcome such issues. The modification of chitosan with PEG prevents interaction with blood components and plasma proteins, reduces interaction with opsonins, improves the solubility of chitosan, and ultimately prevents the removal of nanoparticles from the RES. PEG is an ideal polymer that is highly soluble in water as well as inorganic solvents. It also shows good biocompatibility and biodegradability and low cytotoxicity [12].

PEG is known for its ‘stealth behavior’ and has the ability to be modified with a large number of functional groups on its terminal end groups. It is an FDA-approved linear polyether compound that is nonimmunogenic, nontoxic, and non-antigenic and exhibits very low protein adsorption compared to any other polymer used in drug delivery systems. PEGylated chitosan is obtained by attaching PEG molecules to various reactive groups present on the backbone of chitosan. The modification of chitosan with PEG improves the pharmacokinetic and pharmacodynamic properties of the drug delivery system prepared with PEGylated chitosan derivatives, specifically enhancing body residence time, bioavailability, and water solubility while reducing renal clearance and cytotoxicity [13]. 

The aforementioned problems and reasoning associated with the choice of drug, polymer, delivery systems, and functional groups has led to the decision to formulate nanoparticles with this particular combination of materials and methods, i.e., PEGylated chitosan nanoparticles of TAF, to achieve the best possible therapeutic effect of the drug backed by a strong, biocompatible, and nontoxic drug delivery system. This study aims to synthesize PEGylate chitosan by the initial protection of its amine groups with phthalic anhydride, followed by the deprotection and chemoselective conjugation of PEG to the hydroxyl groups. The drug-loaded PEGylated chitosan nanoparticles were subjected to characterization tests to assess their potential for the successful drug delivery of TAF.

## 2. Materials and Methods

### 2.1. Materials

Tenofovir Alafemaide was received as gift from GENIX PHARMA PVT. LTD., Pakistan. High-molecular-weight chitosan (>75% deacetylated, MW: 310,000–375,000 Da, 800–2000 cP, CAS number: 9012-76-4) was purchased from SigmaAldrich, Burlington, MA, USA. Polyethylene glycol (PEG) 6000 was purchased from Fluka Chemie GmbH, Germany. Thionyl chloride (SOCl_2_, ≥99%), anhydrous tetrahydrofuran (THF, ≥99%, CAS number: 109-99-9), sodium tripolyphosphate (TPP, 85%, CAS number: 7758-29-4), phthalic anhydride (≥99%, CAS number: 85-44-9), sodium hydride (NaH, 60% dispersion in mineral oil, CAS number: 7646-69-7), hydrazine monohydrate (98%, contains 64% hydrazine, CAS number: 7803-57-8), anhydrous N,N-dimethylformamide (DMF, 99.8%, CAS number: 68-12-2), and anhydrous sodium hydroxide pellets (NaOH, ≥98%, CAS number: 1310-73-2) were purchased from Merck & Co., Inc., New York, USA, and pyridine (99.5%) was purchased from Riedel-de Haen. All reagents and solvents used in the synthesis process were of analytical grade. Deionized and double-distilled water was taken from a research laboratory of the University of Central Pharmacy, Pakistan.

### 2.2. Method for Synthesis of PEGylated Chitosan

The PEGylated chitosan was synthesized using the modification of chitosan through hydroxyl groups. In the first step, the deacetylation of chitosan was performed using NaOH, followed by the phthylation of deacetylated chitosan using DMF (step 2). In the third step, the chlorination of phthylated chitosan was achieved by using SOCL_2_, and PEGylated phthylated chitosan was achieved in the PEG activation step (step 4). In the last step, PEGylated chitosan was synthesized for further use in the fabrication of nanoparticles. 

The deacetylation of chitosan was achieved by preparing a 40% (*w*/*v*) solution of NaOH and added the required amount of chitosan powder to it. The chitosan-containing solution was stirred for 4 h at 110 °C in a nitrogenous environment. The uniform solution was filtered and a 40% NaOH solution was added again under same conditions. The final solution was freeze-dried at −50 °C in a lyophilizer.

After the deacetylation of chitosan, the phthylation of chitosan was achieved by taking deacetylated chitosan in a beaker and adding a freshly prepared phthalic anhydride solution. The phthalic anhydride solution was prepared by adding 2.76 g of phthalic anhydride to 20 mL of DMF. The solution was stirred for 8 h at 120 °C in a nitrogenous environment. The resulting product was then cooled at room temperature (25 °C), and ice-cold water was added in the resultant to ensure the precipitation of phthylated chitosan. The precipitates were filtered, washed with methanol to remove unreacted chitosan, and dried overnight.

The PEGylated chitosan was synthesized chemoselectively, where phthylated chitosan was attached to PEG after the activation of PEG with NaH (used as a catalyst to make a conjugate of chitosan and PEG). The synthesis of PEGylated chitosan was completed in substeps. First, phthylated chitosan was added to 20 mL of pyridine, and SOCL_2_ was added to the resultant solution in a volume ten times greater than that of chitosan. The reaction was stirred for 30 min at 80 °C in a nitrogenous environment. After stirring, the resultant solution was cooled at room temperature, and chilled water was added to the solution for precipitation. The solution was filtered and dried to yield chlorinated phthaloyl chitosan. In the next step, the desired amount of PEG was taken, added to the already prepared NaH solution after mixing with anhydrous THF, and stirred for two hours at 60 °C in a nitrogenous environment. The previously prepared chlorinated phthylated chitosan was added to the reaction mixture and again stirred for 16 h. The reaction mixture was allowed to cool, and methanol was added to make precipitates. The mixture was filtered and dried to obtain PEGylated chitosan. 

**Figure 1:**
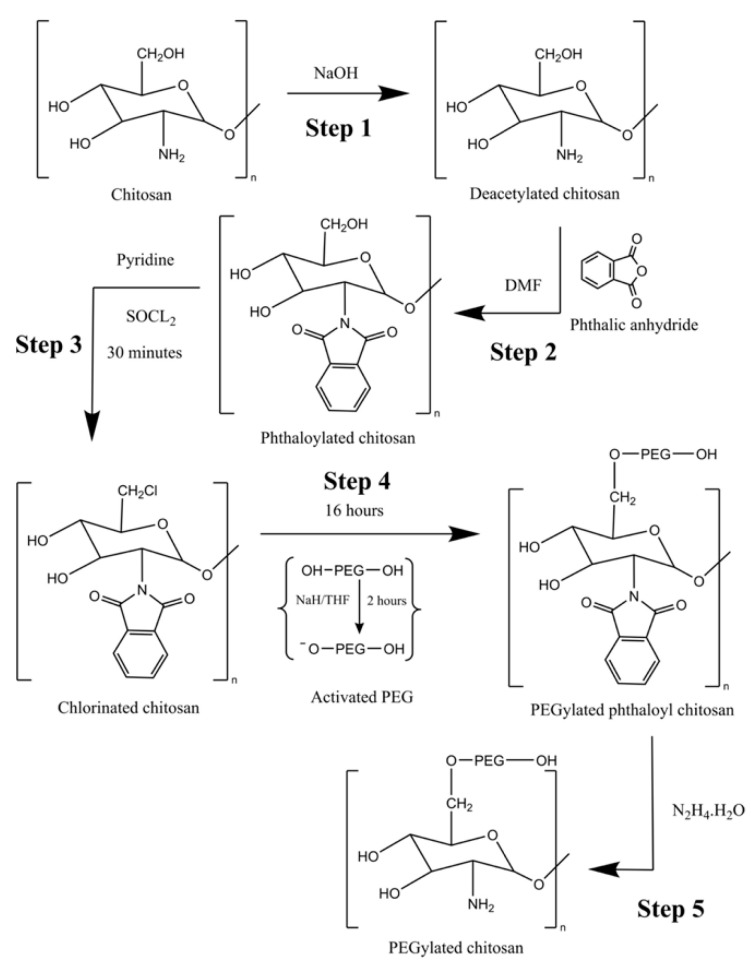
Reaction scheme of PEGylated chitosan.

After the deacetylation of chitosan, the protection of the amine group was ensured, and in the last step, the deprotection of PEGylated phthylated chitosan was achieved by taking 15 mL of hydrazine monohydrate along with 30 mL of distilled water and mixing for 16 h at 100 °C under constant magnetic stirring until a viscous solution was left in the beaker. The same procedure was repeated three times by reconstituting the beaker contents with distilled water until a solid residue remained. The solid residue was vacuum-dried to obtain the desired PEGylated chitosan and was stored for further use. The complete synthesis process is presented in Figure 1.

### 2.3. Method for Preparation of PEGylated Chitosan TAF Nanoparticles

**Figure 2:**
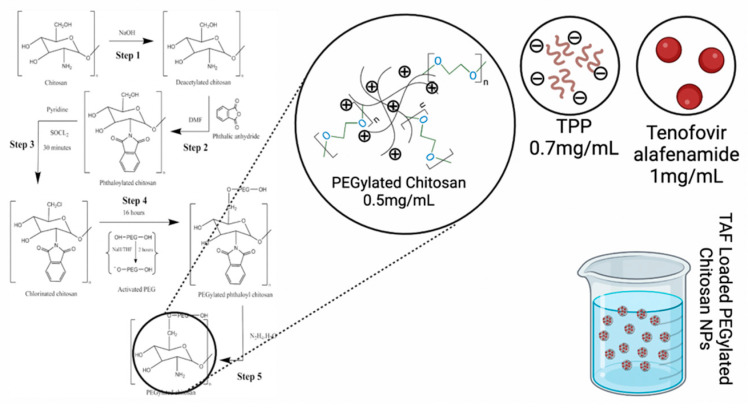
Graphical representation of the preparation of TAF nanoparticles.

An ionic gelation method was used to prepare PEGylated chitosan nanoparticles because it was extensively used in the literature for the preparation of polymeric nanoparticles. First, the polymer was dissolved in a 1% (*v*/*v*) acetic acid solution, and a pH of 5 was maintained using NaOH or HCL as required. The TPP solution was prepared after dissolving TPP in double-distilled water to obtain a concentration of 0.7 mg/mL and a pH of 3 was maintained using NaOH or HCL as required. The polymer solution was stirred on a magnetic stirrer for one hour at room temperature, and a dropwise TPP solution was added at a constant speed to make polymeric nanoparticles. The prepared nanoparticles were centrifuged at 14,000 rpm and 5 °C for 30 min. Then, the supernatant and sediment were separated. The sediment was redispersed using deionized water and characterized by a particle size analyzer for the particle size, zeta potential, and polydispersity index. A graphical representation of the preparation of nanoparticles is presented in Figure 2.

### 2.4. Characterization 

The physicochemical properties of the synthesized polymer and fabricated nanoparticles were checked to confirm the successful synthesis of the polymer and the potential of the nanocarrier system for drug delivery. 

#### 2.4.1. Determination of λ_max_ and Calibration Curve of TAF

The wavelength of a substance that shows the maximum absorption is called the λ_max_. This value is usually calculated with a UV-visible spectrophotometer after preparing drug solutions. A solution of TAF was prepared by dissolving the drug in distilled water and making up the volume, and wavelength scans were carried out on a spectrophotometer. The calibration curve of TAF was determined by making serial dilutions of the drug from a stock solution in a distilled water and PBS buffer solution. The stock solution was prepared by dissolving 10 mg of drug in 100 mL of distilled water to obtain a concentration of 0.1 mg/mL. The first dilution was prepared by taking 2 mL of stock solution and adding distilled water up to 10 mL. The second dilution was prepared by taking 4 mL from the stock solution and making up to 10 mL with distilled water. The next dilutions were prepared by taking 6 mL from the stock solution, and distilled water was added up to 10 mL. The absorbance values of all prepared dilutions were checked with a UV-visible spectrophotometer at the wavelength determined in the previous step.

#### 2.4.2. Fourier-Transformed Infrared (FTIR) Spectroscopy

FTIR studies were conducted to determine the compatibility of the drug with other formulation excipients. FTIR spectroscopy is sensitive to the chemical surface of nanoparticles and helps in the identification of the functional groups and bonds present in the drug and other excipients. [14]. FTIR was performed on all steps during the synthesis of PEGylated chitosan, the pure drug, polyethylene glycol, chitosan, and drug-loaded nanoparticle formulation using an Agilent Cary 630 FTIR spectrometer (California, USA).

#### 2.4.3. Scanning Electron Microscopy (SEM)

SEM studies were conducted to check the surface morphology of the polymer and the size of the nanoparticles [15]. The SEM studies were carried out using a Zeiss EVO LS10 (Carl Zeiss, Oberkochen, Germany).

#### 2.4.4. X-ray Diffraction (XRD) Analysis

An XRD analysis was conducted to analyze degree of crystallinity of the structures of the polymers and prepared formulations. It helped to evaluate the impact of changes on the crystallinity of formulations [16]. The XRD analysis was carried out with a Bruker D8 Discover diffractometer (Massachusetts, USA).

#### 2.4.5. Particle Size Analysis

The nanoparticles were characterized for physical parameters such as the particle size, zeta potential, and polydispersity index (PDI) using a Zetasizer Nano ZS (Malvern, UK). The nanoparticles were checked in a standard range for nanoparticles, which is 1–1000 nm size. The zeta potential is the measure of the surface charge of nanoparticles. It is measured to assess the degree of stability of nanoparticles in a liquid formulation, and the magnitude of the zeta potential indicates the degree of repulsion between adjacent nanoparticles with similar charges within a liquid. The PDI is a measure of the standard deviation from the average particle diameter within a particle solution. It is measured to check the uniformity of a solution in terms of particle size [17]. Physical characterization was carried out for all prepared nanoparticle formulations in order to determine the above-mentioned physical parameters.

#### 2.4.6. Drug-Loading Capacity

The drug-loading capacity (DLC) is a measure of the amount of drug that is loaded compared to the total weight of the nanoparticles. It determines what percent of the nanoparticle mass comprises the encapsulated drug [18]. The percent DLC was determined for drug-loaded nanoparticles. To determine the % DLC, the nanoparticle formulations were subjected to centrifugation at 14,000 rpm for 30 min. After centrifugation, the supernatant liquid was used to check the absorbance value in a UV spectrophotometer at a wavelength of 262 nm. This value was put into the calibration curve regression equation to find the amount of drug present in the supernatant liquid, from which the drug-loading capacity was determined using the following formula:

$$Drug\;loading\;capacity=\frac{Total\;drug-unentrapped\;drug}{Total\;weight\;of\;nanoparticles}\times 100$$

#### 2.4.7. Entrapment Efficacy

The entrapment efficiency is the measure of the amount of drug entrapped in the nanoparticles compared to the total amount of drug subjected to loading into nanoparticles. It determines what percentage of the total added drug was entrapped into the nanoparticles, thus determining the efficiency of the nanoparticles in terms of entrapment [19]. The %EE was determined for drug-loaded nanoparticles. For this purpose, the amount of unentrapped drug in the nanoparticles was determined by separating the nanoparticles from their aqueous medium by centrifugation at 14,000 rpm for 30 min. The supernatant obtained after centrifugation was evaluated in a UV-visible spectrophotometer. The resulting absorbance value was put into the regression equation obtained from the calibration curve of TAF to determine the amount of unentrapped drug in the nanoparticles. Then, the %EE of the nanoparticles was determined using the following formula:

$$Entrapment\;efficacy=\frac{Total\;drug-unentraped\;drug}{Total\;drug}\times 100$$

#### 2.4.8. In Vitro Drug Release

For in vitro drug release studies, a 12 kD dialysis membrane was used to assess the release of TAF from PEGylated chitosan nanoparticles and non-PEGylated nanoparticles using a USP drug dissolution apparatus II (Paddle). A 1 mL nanoparticle solution was mixed with a 4 mL pH 7.4 PBS solution of in dialysis membrane bag. The membrane bag was tied with the paddle of the dissolution apparatus. The paddle was immersed in 500 mL of dissolution medium with the temperature set at 37 °C and a speed of 50 rpm. Then, 5 mL of sample solution was removed every hour for 9 h and at 24 h, 47 h, and 48 h, with 5 mL of fresh dissolution medium added after every sample. The samples were run in a UV-visible spectrophotometer at 262 nm. Absorbance was noted, and the % drug release was calculated using the following equation [20]:

$$\%\;Drug\;Release=\frac{Absorbance\;of\;sample}{Absorbance\;of\;standard}\times 100$$

#### 2.4.9. Drug Release Kinetic Modeling

Drug release kinetic studies were performed to check the pattern of TAF release from the nanoparticles. To check this, zero-order, first-order, Higuchi’s, Hixson–Crowell, and Korsmeyer–Peppas models were generated in DD Solver software. In the Korsmeyer–Peppas model, *n* was also calculated to check the diffusion pattern of the drug from the carrier. The drug followed a Fickian diffusion mechanism if the n value was less than 0.45; otherwise, the mechanism was non-Fickian (*n* > 0.45). The drug release data from all formulations of the nanoparticles were subjected to the following equations:

Zero-order equation:

$$F=k_{o}\times t$$

where *t* is time and *k_o_* is the release rate constant for zero-order kinetics;

First-order equation:

$$F=100\times \left[1-Exp\left(-k_{1}\times t\right)\right]$$

where *k*_1_ is the release rate constant for the first-order kinetics; 

Higuchi’s equation:

$$F=k_{H}\times t^{0.5}$$

where *k_H_* represents the Higuchi release rate constant; 

Hixson–Crowell equation:

$$F=100\times \left[1-{\left(1-k_{HC}\times t\right)}^{3}\right]$$

where *k_HC_* is the Hixson–Crowell rate constant; 

Korsmeyer–Peppas equation:

$$F=k_{KP}\times t^{n}$$

where *k_KP_* is a constant corresponding to the structural and geometric characteristics of the device and *n* is the release exponent, which is indicative of the mechanism of the drug release [21]. The R^2^ values of all the models and the n value from the Korsmeyer–Peppas model obtained from the results of DD solver were assessed to determine which model best fit the mechanism of drug release from the nanoparticles.

## 3. Results and Discussions

### 3.1. Determination of Absorption Spectra of TAF

**Figure 3:**
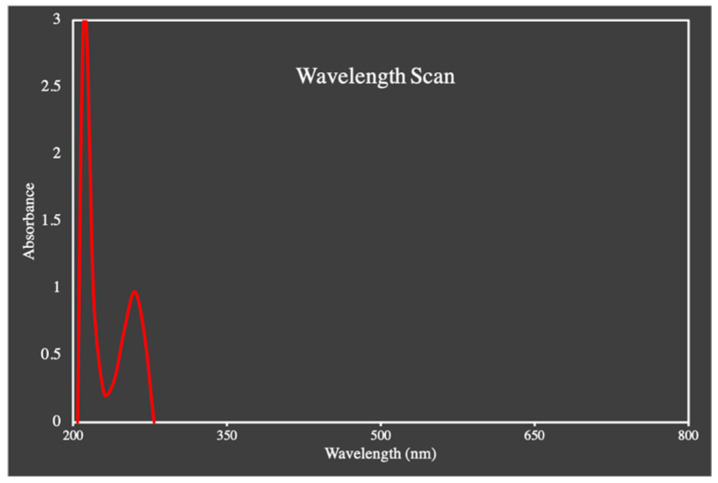
The absorption spectra of TAF.

The absorption spectra of TAF were calculated from UV-visible spectrophotometer via a wavelength scan. The figure showed the maximum absorbance at 260 nm, and in the literature it was reported as 262 nm. The obtained wavelength is presented in Figure 3.

### 3.2. Determination of Calibration Curve of TAF

**Figure 4:**
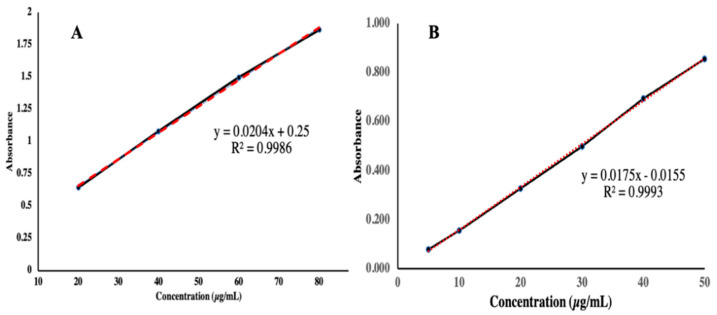
Calibration curve of TAF in (**A**) distilled water and (**B**) PBS buffer solution.

The calibration curve of TAF was determined according to the method mentioned in the Materials and Methods Section. The absorbance was determined for all dilutions at a λ_max_ of 262nm. The absorbance values of all dilutions with different concentrations in distilled water as well as a PBS buffer of 7.4 pH are presented in Figure 4.

### 3.3. Chemical Compatibility Studies

#### 3.3.1. FTIR of Synthesized Polymer

**Figure 5:**
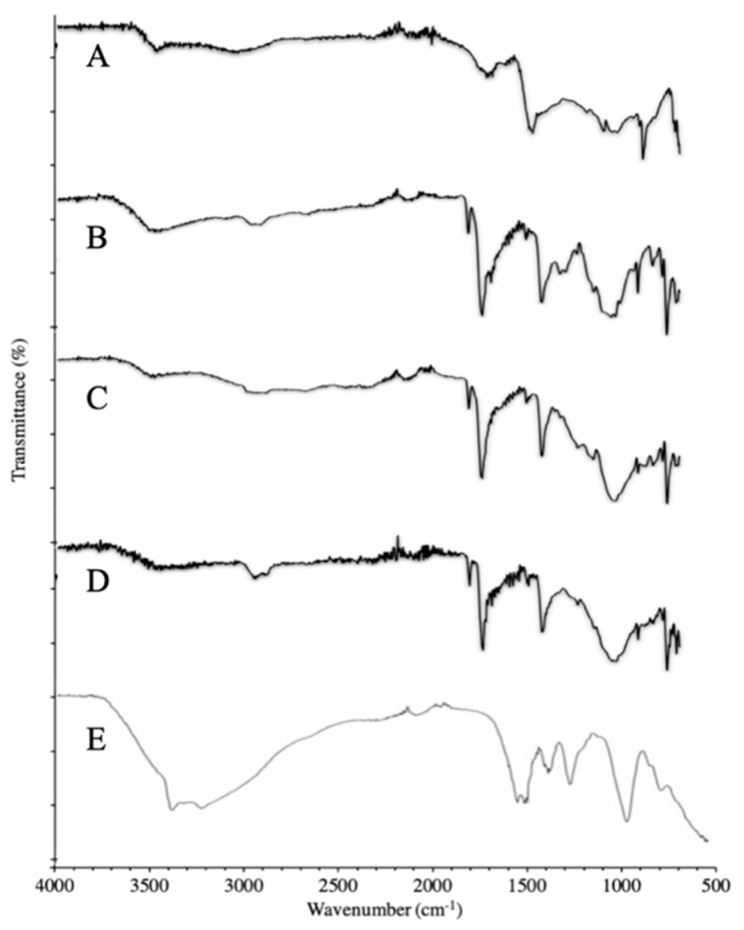
FTIR analysis of synthesized PEGylated chitosan. (**A**) Deacetylated chitosan. (**B**) Phthaloylated chitosan. (**C**) Chlorinated chitosan. (**D**) PEGylated chitosan. (**E**) Deprotected PEGylated chitosan.

The FTIR analysis was conducted on all steps of polymer synthesis. The first step was the deacetylation of chitosan. In Figure 5A, the FTIR of deacetylated chitosan shows a prominent band in the range of 3000–3500 cm^−1^, which corresponds to the N-H and O-H stretching vibrations. The peaks at 2950.18 cm^−1^ and 2847.86 cm^−1^ represent C-H symmetric and asymmetric vibrations, respectively. These bands are characteristic bands of polysaccharides that represent the high linkage prevalence of C-H bonds and are exhibited in the spectra of many other polysaccharides such as carrageenans, xylan, and glucans [22]. C=O amide I stretching, the N-H bending of amide II, and C-N amide III stretching are represented by the bands at 1727.62 cm^−1^, 1574.80 cm^−1^, and 1366.06 cm^−1^, respectively, which are indicative of the residual N-acetyl groups that were left after deacetylation. The band at 1149.88 cm−1 represents the asymmetric stretching vibrations of the C-O-C bridge. CH_2_ bending vibrations are depicted by the peak at 1433.16 cm^−1^. The peaks at 1060.42 cm^−1^ and 1026.88 cm^−1^ exhibit the C-O stretching and bending vibrations. The FTIR of chitosan showed the absence of the acetyl (CH_3_) peaks at 1375 cm^−1^, which were reported by Queiroz et al. in their study [23]. This confirms the successful deacetylation of chitosan by treating it with a NaOH solution. However, the FTIR spectrum of deacetylated MMWC showed some bands at 1718.30 cm^−1^, 1578.52 cm^−1^, and 1423.84 cm^−1^, which are indicative of the residual N-acetyl groups that were left after deacetylation. Similar results were reported by Silva et al., who prepared multifunctional chitosan and gold nanoparticles and observed bands of residual n-acetyl groups in the FTIR spectrum of deacetylated chitosan [24]. In Figure 5B, the FTIR of phthaloylated chitosan shows a prominent band in the range of 3200–3400 cm^−1^, which corresponds to the N-H and O-H stretching vibrations. The successful phthaloylation of deacetylated chitosan was confirmed by the appearance of the peaks at 1772.34 cm^−1^ and 1705.25 cm^−1^, corresponding to the C=O imide bond of the phthaloyl group in the FTIR spectrum of phthaloylated MMWC, and at 1774.21 cm^−1^ and 1701.52 cm^−1^ in the FTIR spectrum of phthaloylated HMWC. A study conducted by Malhotra et al. reported similar results, where the imide bond could be observed at 1774 cm^−1^ and 1702 cm^−1^ in the FTIR spectrum of phthaloylated chitosan [25]. In Figure 5C, phthaloylated chitosan was chlorinated, and the successful completion of the chlorination process was confirmed by the appearance of a peak at 719.37 cm^−1^, which represented the formation of an organic halogen bond within the structure of chitosan, which is in line with the results reported by Najafabadi et al. [26]. Furthermore, the reduction in the magnitude of the peaks from 3200–3400 cm^−1^ shows the reduction in O-H groups in chlorinated phthaloyl chitosan. In Figure 5D, the peak at 3410.14 cm^−1^ exhibits C-H stretching vibrations of PEG. The PEGylation of chitosan was confirmed by the appearance of characteristic peaks of PEG at 1386.56 cm^−1^, 1194.61 cm^−1^, 989.60 cm^−1^, and 874.06 cm^−1^. C-O stretching vibrations are presented by the peak at 1011.97 cm^−1^, and the peak at 1105.55 cm^−1^ shows the C-O-C bridge of chitosan. In Figure 5E, the FTIR spectrum of deprotected PEGylated chitosan shows O-H stretching vibrations at 1062.29 cm^−1^, which represent the characteristic peaks of PEG, confirming the PEGylation of chitosan. The peaks at 1608.34 cm^−1^, 1576.66 cm^−1^, and 892.69 cm^−1^ correspond to the amide I, amide II, and pyranose structures, respectively, which are the main peaks of the chitosan molecule. The deprotection of the sample was confirmed by the presence of a C=O imide peak at 1710 cm^−1^, which came from the hydrazine used during deprotection. The results were similar to the study reported by Malhotra et al., where they prepared peptide-tagged PEGylated chitosan nanoparticles for siRNA delivery [27].

#### 3.3.2. FTIR of Synthesized NPs

**Figure 6:**
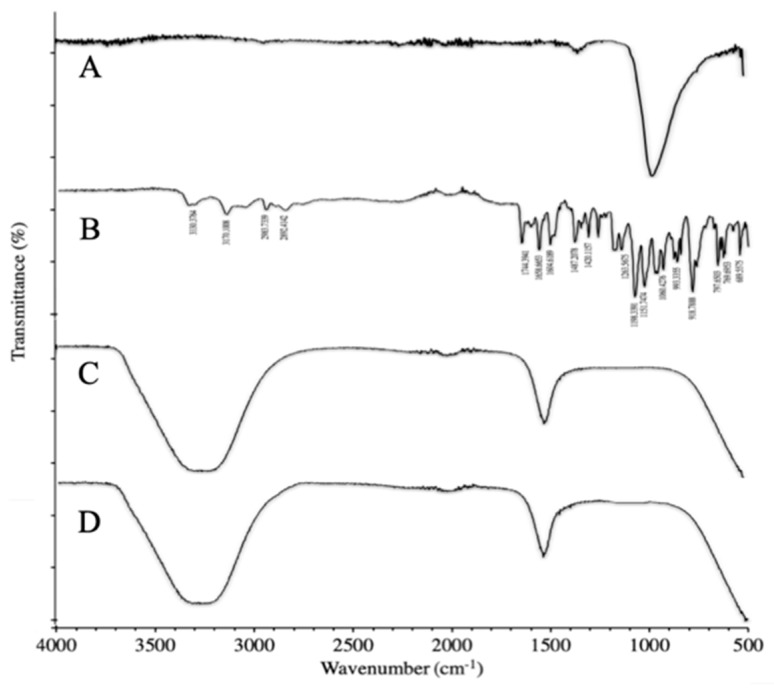
FTIR analysis of synthesized PEGylated chitosan. (**A**) TPP. (**B**) TAF. (**C**) Deprotected PEGylated chitosan nanoparticles. (**D**) TAF-loaded PEGylated chitosan nanoparticles.

In Figure 6A, the FTIR spectrum of TPP shows a peak at 1470.43cm−1 corresponding to P=O stretching vibrations. PO_3_ symmetric and asymmetric stretching vibrations are shown by the peak at 1455.52 cm^−1^. The antisymmetric stretching vibrations of P-O-P bridge are exhibited by the peak at 1092.10 cm^−1^. In Figure 6B, the FTIR spectrum of TAF shows a weak N-H band at 3330.38 cm^−1^ and a peak of aromatic stretching vibrations at 3170.10 cm^−1^. The C=O and P=O stretching vibrations are depicted by the peaks at 1744.39 cm^−1^ and 1658.66 cm^−1^, respectively. The NH_3_ scissoring band stretching vibrations are exhibited by a sharp peak at 1604.61 cm^−1^. The peaks at 1420.11 cm^−1^ and 1487.20 cm^−1^ correspond to the aromatic CN stretching vibrations in pairs. The deformation of C-N is represented by the peak at 1263.56 cm^−1^. The band from 600 cm^−1^ to 900 cm^−1^ corresponds to the C-H out-of-plane deformation, and lastly the peak at 918.78 cm^−1^ exhibits the N-H wagging band. In Figure 6C, the deprotected PEGylated chitosan was further treated with TPP to formulate nanoparticles. The FTIR spectrum of nanoparticles depicts very sharp peaks for N-H and O-H stretching vibrations within the range of 3200–3500 cm^−1^, which are suggestive of the stronger hydrogen bonding within the nanoparticles. Moreover, a hypochromic shift in the peaks of the CONH_2_ and NH_2_ vibrations is observed in the nanoparticles compared to those of simple deacetylated chitosan, which is a result of the interaction between the NH_3_ of chitosan and the phosphate groups of the TPP used during nanoparticle formulation, which coincides with the results of a study reported by Qureshi et al. [28]. In Figure 6D, the FTIR spectrum of TAF-loaded PEGylated chitosan nanoparticles shows the same characteristic peaks as that of chitosan nanoparticles. The absence of characteristic peaks of TAF shows that TAF was fully encapsulated without any chemical interaction. The spectrum shows all the peaks of PEGylated chitosan nanoparticles. However, none of the peaks of TAF appear in the FTIR of the drug-loaded nanoparticles. Similar results were reported by Ulu et al. in their study where they proposed that the absence of peaks of TAF suggests that the drug was fully encapsulated within the nanoparticles and no possible interaction of the drug with the components of the nanoparticles was observed, which is why only the peaks of PEGylated chitosan nanoparticles were observed and the peaks of TAF were masked within the nanoparticle structure [29].

### 3.4. XRD Studies

**Figure 7:**
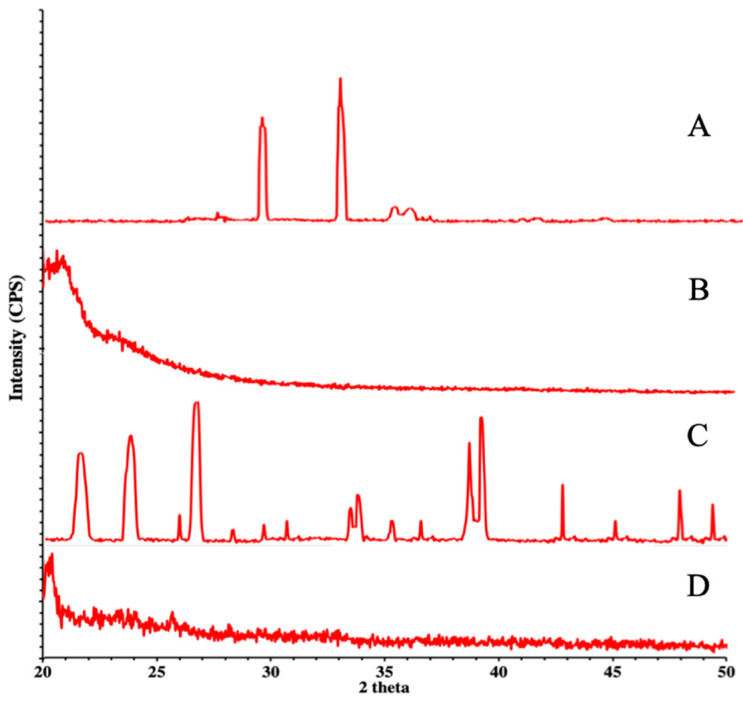
Illustration of XRD of PEG (**A**), PEGylated chitosan (**B**), the drug (**C**), and TAF PEGylated nanoparticles (**D**).

X-ray diffraction studies were performed on pure PEG, PEGylated chitosan, TAF, and the TAF-loaded NP formulation. The obtained diffractogram of PEG showed high-intensity sharp peaks at 19° and 24° as well as minor peaks at 27° and 28°, which confirmed the crystalline structure of PEG (Figure 7A). The PEGylated chitosan polymers of different molecular weights showed amorphous structures with zero peaks (Figure 7B). The pure TAF was a crystalline powder with many high-intensity sharp peaks at different points as well as small peaks indicating the various functional groups of the drug (Figure 7C). The diffractograms of the drug-loaded nanoparticles (Figure 7D) showed no prominent peaks. However, diffused peaks were observed, which depicted the amorphous form of the prepared formulation. The absence of sharp peaks of the drug in the XRD of formulated nanoparticles can be attributed to the use of various chemicals during the formation of the nanoparticles, which resulted in the amorphization process, causing the disappearance of characteristic peaks of the crosslinked polymer and drug. Similar results were reported by Melo et al. [30] during the preparation of chitosan/PEG nanoparticles loaded with indole-3-carbinol and by Maity et al. [31] during the study of naringenin-loaded alginate-coated chitosan core–shell nanoparticles, where they observed the disappearance of characteristic peaks in the drug-loaded nanoparticles. Thus, it is possible to consider that the disappearance of the drug peaks in the nanoparticles is a clear indication of the dispersion, at the molecular level, of the drug within the nanoparticle matrix.

### 3.5. SEM Analysis 

**Figure 8:**
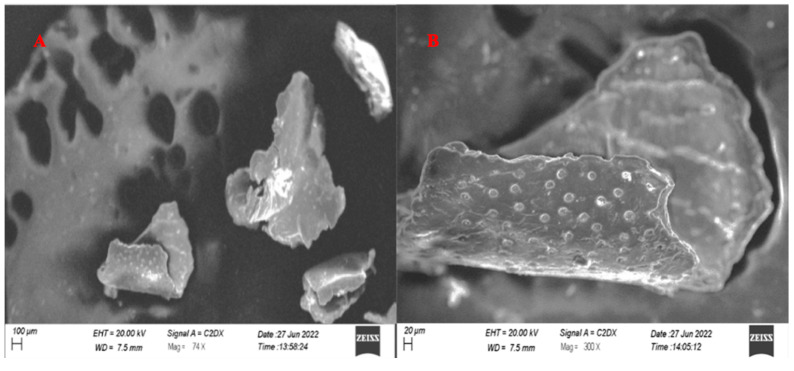
SEM image of synthesized PEGylated chitosan at (**A**) 74× and (**B**) 300×.

**Figure 9:**
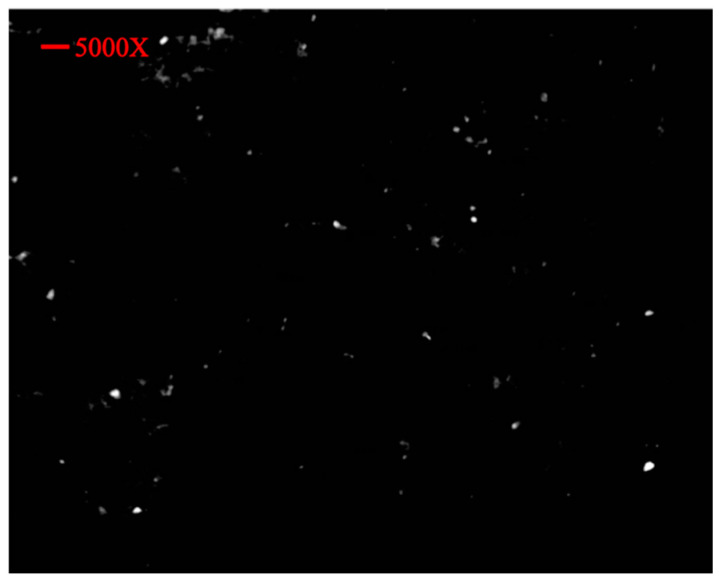
SEM image of PEGylated chitosan nanoparticles at 5000×.

Scanning electron microscopy was performed on the PEGylated polymer (Figure 8) and the nanoparticle formulation (Figure 9). The SEM images showed that the polymer particles were rectangular to cylindrical in shape, along with spherical and irregularly shaped particles. With such different results, it was difficult to claim the best structural morphology of the synthesized polymeric particles. In the SEM images, it was also revealed that the particles had slightly spongy as well as amorphous outer surfaces. In the XRD diffractogram, the synthesized polymer had an amorphous nature, and the SEM results were authenticated with XRD where distinct or sharp peaks were observed. Ulu et al. developed simple and drug-loaded chitosan nanoparticles and reported that the nanoparticles were roughly spherical in shape and evenly distributed [29]. The spherical shape of the nanoparticles was due to the tendency of chitosan molecules to fuse with each other with extensive hydrogen bonds in the structure [32]. The current results correlate with previous studies where similar results were reported [33,34]. Additionally, Ulu et al. also reported that TAF loaded in nanoparticles did not change the morphology of fabricated nanoparticles [29]. The PEGylation of chitosan and drug-loaded PEGylated nanoparticles was performed, and the results of the FTIR and SEM analyses authenticated the successful synthesis of the polymer and nanoparticles. For further identification to confirm the drug loading, XRD was performed on the pure drug, chitosan, PEG, PEGylated chitosan, and prepared nanoparticles. The XRD spectra of PEGylated chitosan nanoparticles showed an amorphous nature, which confirmed that the crystal structure of the drug was tapered. 

The SEM images of nanoparticles showed well-segregated particles, as authenticated by the particle size analyzer results. The particles in the SEM images showed the successful fabrication of nanoparticles with PEGylated chitosan and advocate for the stability of nanocarriers. The figure illustrates the spherical shape of nanoparticles and the uniform distribution. From the SEM images, it can be observed that the particles were of a nanosized range that was also studied or reflected by the zeta sizer outcomes. The well-segregated particles might be due to a considerable charge on the particle surfaces that was observed in the zeta potential analysis.

### 3.6. Drug-Loading Capacity and Entrapment Efficacy of NPs

The %EE of TAF loaded in PEGylated chitosan nanoparticles was calculated to be ~90% and ~15%. Ulu et al. aimed to develop TAF loaded in simple chitosan nanoparticles and found an EE of approximately 50% [29]. In another study, Shailender et al. found an EE of 48.2%, but they prepared nanoparticles of chitosan loaded with a drug that was similar to that used in the current project [35]. The literature reported the effect of the molecular weight of the polymer and found that the EE and molecular weight have a direct relation with each other [36]. In this study, we also reported the same results, where EE increased with an increase in the molecular weight of the polymer. These findings can be attributed to the idea that an increased molecular weight of the polymers results in a more porous or expanded nanoparticle matrix, allowing more drug to enter and encapsulate. The results were authenticated in the literature, where Meng et al. prepared tenofovir nanoparticles using different molecular weights of chitosan (low, medium, and high) and reported less EE, but with an increase in the molecular weight, the EE surged [37]. Therefore, the % of the drug loading into the chitosan-based nanoparticles might involve different mechanisms, such as adsorption, encapsulation, and electrostatic interaction [36]. Therefore, it can be hypothesized that encapsulation occurs due to the physical confinement of the drug in the interstices of the nanostructured material during the gelation process by various interactions or due to hydrogen bonding between the drug and the polymers [38]. Ideally, an efficient drug delivery system would possess an excellent degree of drug association. A high EE was possible due to the hydrophobic nature of drugs, which helps the drug to enter the nanocarrier system. In this study, TAF showed good EE and indicated ionic interactions between the polymer and TPP. This interaction ultimately resulted in the preparation of highly entrapped drug nanoparticles. In a previous study conducted by Shahid et al., they prepared ticagrelor-loaded simple chitosan and ticagrelor-loaded thiolated chitosan nanoparticles and reported EE values of 84.1% and 94.3%, respectively [39]. In another study, Bo Fan et al. reported an EE of 98% in thiolated chitosan nanoparticles [40].

### 3.7. Particle Size Determination

**Figure 10:**
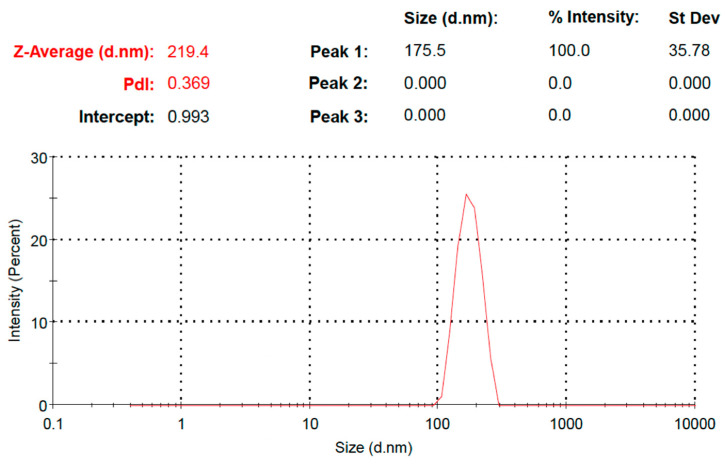
Particle size distribution of TAF PEGylated chitosan nanoparticles.

**Figure 11:**
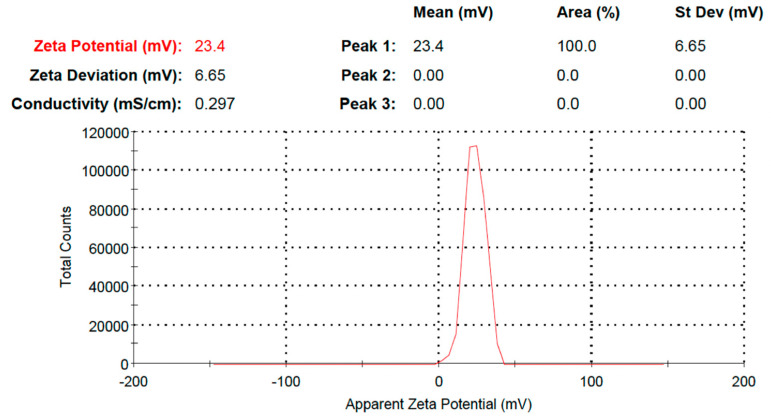
Zeta potential of TAF PEGylated chitosan nanoparticles.

The particle size of the drug-loaded PEGylated chitosan was analyzed, and the observed particle size distribution was 219.4 nm with a PDI of 0.369 (Figure 10), which indicated the stability of the nanoparticles (Figure 11). Ulu et al. checked the hydrodynamic diameters of drug-unloaded and drug-loaded nanoparticles and found average particle sizes of 200 to 340 nm [29]. The increase in the average particle size of nanoparticles might be due to drug incorporation between the chitosan network [32]. The zeta potential was 23.4mV (Figure 11). The results were similar to the study reported by Ulu et al., where a higher zeta potential of 42.8 mV was observed for chitosan due to its cationic nature [29,41]. In other studies, Yien et al. [42] and Shailender et al. [35] prepared drug-loaded chitosan nanoparticles and reported higher zeta potential values. Similar results were reported by Machado et al., where they fabricated composites of nanoparticles (tenofovir-loaded poly(lactic-co-glycolic acid)/stearylamine) [43]. The PDI was also checked for the prepared nanoparticles, where a PDI of 0.369 was observed in the prepared formulation. In a previous study, a low PDI was observed (0.44) [29], and a lower PDI indicated the fabrication of stable nanoparticles [35]. However, a higher PDI was also observed in a previous study where they reported a PDI of 0.65 for TAF-loaded chitosan nanoparticles [44]. The PDI in the current study showed a uniform distribution. However, an increased PDI supports the good colloidal nature and long-term stability of nanoparticles [29]. Our results were comparable with many studies, such as Mariadoss et al. [45], Zhang et al., [46] and Ulu et al. [29].

### 3.8. In Vitro Drug Release

**Figure 12:**
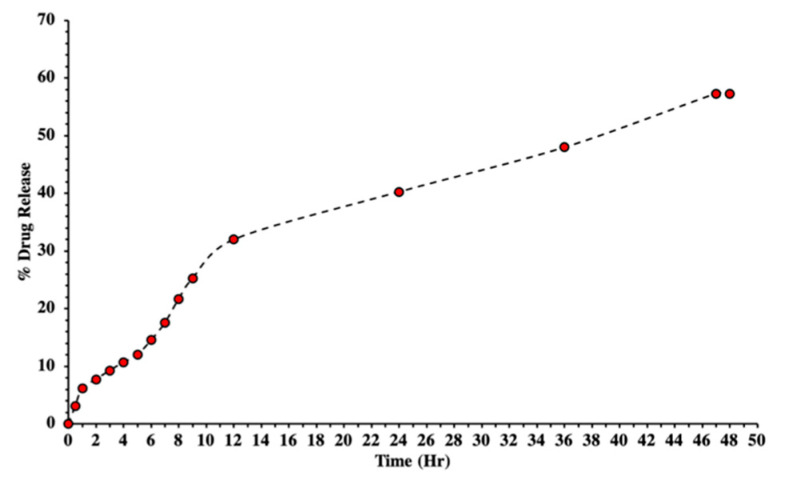
In vitro percent of drug release from PEGylated chitosan nanoparticle formulations.

The in vitro % drug release of all formulations was carried out in USP Dissolution apparatus II using a dissolution medium (PBS) with a pH of 7.4. The % of the drug released from the nanoparticles was calculated for 48 h (Figure 12). Approximately 60% of the drug was released from the PEGylated nanoparticle formulation. The TAF was released from the prepared biodegradable polymeric nanocarrier system from the surface because of desorption, erosion, degradation, reabsorption, and diffusion mechanisms of the polymeric network [45,47]. A study conducted by Ulu et al. reported a burst release of approximately 53% in a day [29], but in our study almost 35% of the drug was released in first 24 h. The burst release was due to the higher amount of drug dispersed on the surface or near the surface of the nanocarrier system [36]. After two days, approximately 60% of the drug was released from the nanoparticles. These results were in agreement with the previous study where Meng et al. reported almost complete drug release from nanoparticles using low-molecular-weight chitosan within 48 h and 50–70% from high- and medium-molecular-weight chitosan within 120 h [37]. In addition, 80–90% release was reported by Ulu et al. [29], and 80% release within 72 h was reported by Zhang et al. [48]. Another study prepared thiol-modified chitosan nanofibers to check the release of tenofovir [49]. They reported an 80–100% release from the carrier within 50 h. To summarize, we can say that our release outcomes corresponded with the literature. 

Polymers such as PEG have the capacity to solubilize in water as well as organic solvents, which is extensively used in grafting many polymers. It is biocompatible and biodegradable and has no immunogenicity, antigenicity, or toxicity [25]. In 2004, Gorochovceva and Makuska proposed a novel method for the synthesis of PEGylated chitosan by modifications at hydroxyl groups [50]. Previously, PEG was extensively used as a cross-linker for forming graft polymers to enable drug release [51]. Different techniques were used by changing parameters to prepare a nanocarrier system of modified chitosan. Those parameters were the concentration of the polymer, the crosslinker, the pH, the molecular weight, the drug-to-polymer ratio, the surfactants, and the stabilizers. However, all those parameters directly or indirectly alter the structure as well as the morphology of prepared chitosan nanoparticles and the release patterns of drugs [52]. Furthermore, drug release was controlled by PEG, and the same pattern was reported in our study, where drug release was decreased and approximately 60% of the drug was released in 2 days. Our results were supported by a similar study conducted by Moradkhannejhad et al., where they checked the curcumin drug release from a nanocarrier system. They reported that with an increase in the PEG content the drug release was improved because of the hydrophilic nature of PEG. They also studied the drug release pattern by varying the molecular weight of PEG and found that the formulation with PEG400 showed more drug release compared to the formulation with PEG6000. This change was due to the hydrophilicity of PEG and its consequent swelling capacity, which ultimately improved drug release. Therefore, we can say that the lower the molecular weight, the higher the hydrophilicity and vice versa [53].

### 3.9. Kinetic Model Studies

For kinetics studies, the results of the % of drug release were added in the DD Solver software to check all the R2 values for kinetic models such as first-order, zero-order, Hixson–Crowell, Korsmeyer–Peppas, and Higuchi (Table 1). The drug-loaded PEGylated chitosan formulation showed first-order kinetics. First-order release indicates that the drug release was dependent on the initial concentration of the formulation. Moreover, the analysis of the R2 values of all kinetics models depicted that our formulation followed the Korsmeyer–Peppas model (R^2^ = 0.9885). The Korsmeyer–Peppas model represents a one-dimensional drug release relative to the drug dosage form [54]. A study conducted by Jusu et al. checked the drug release on all five models that we used in our study and found that all PEGylated drug-loaded microspheres followed the Korsmeyer–Peppas model [55]. In the Korsmeyer–Peppas model, the n value represents the diffusion type. The release may follow Fickian diffusion if the n value is less than or equal to 0.5. Values ranging from 0.5 to 0.85 represent anomalous or non-Fickian diffusion, and values greater than 0.85 refer to a case II transport mechanism [26]. Our formulation showed non-Fickian diffusion with an n value greater than 0.45. A non-Fickian-type release mechanism means that the phenomena responsible for drug release are drug diffusion processes from the NPs coupled to the relaxation of the polymeric chains [56]. Our results corresponded with the study of Jusu et al., where the n value was in range of 0.446 to 0.889, consistent with non-Fickian diffusion or the anomalous transport of drug release that involves two phenomena: drug diffusion as well as drug relaxation from the matrix [55,57]. A similar study was conducted on tenofovir-loaded chitosan nanoparticles and found the best fitting model to be Higuchi (0.9794). This model depicts that liquid penetrates into the nanoparticles and dissolves the drug molecules, and ultimately the release of the drug is predominately controlled by the diffusion mechanism [29].

## 4. Conclusions

We concluded that the successful synthesis of PEGylated chitosan was achieved using a novel approach to form PEG alkoxide using NaH as a catalyst. The synthesized PEGylated polymer has the ability to deliver TAF. The formulated nanoparticles showed a nanosize range for their particle sizes with a polydispersity index of 0.369 and a relatively high entrapment efficiency. The SEM analysis showed that the polymer particles have a rectangular to cylindrical shape along with some spherical and irregularly shaped particles due to which the determination of the exact structural morphology of the particles was complex. The findings of the XRD analysis showed the masking of the sharp peaks of the drug in the XRD of the formulated nanoparticles, indicating amorphization and dispersion, at the molecular level, of the drug in the nanoparticle matrix. The drug release studies showed that approximately 60% of the drug was released from the formulation within 48 h, following the Korsmeyer–Peppas model of drug release. All the findings suggest that the developed nanocarrier system can effectively deliver TAF and in the future can help in providing promising treatment and management of hepatitis B. However, cellular cytotoxicity studies and in vivo analyses are further required to authenticate the formulated nanoparticle system. Therefore, the proposed formulation is a major breakthrough in this field, and a nonviral vector can be further prepared for biomedical applications that involve the target-mediated delivery of drugs.

## Figures and Tables

**Table 1:** Drug release kinetic models.

Formulation		Formulation
Zero-Order	*K*_0_	1.329
	*R* ^2^	0.8693
First-Order	*K*_1_	0.021
	R^2^	0.9557
Higuchi Model	*K_H_*	7.757
	*R* ^2^	0.9588
Korsmeyer–Peppas Model	*K_KP_*	5.066
	*N*	0.632
	R^2^	0.9885
Hixson–Crowell Model	*K_HC_*	0.006
	R^2^	0.9316
Diffusion mechanism		Non-Fickian

## Sample Availability

Samples of the compounds are not available from the authors.
